# Confidential Conversations in Palliative Care: Capability, Vulnerability, and Relational Encounter

**DOI:** 10.1111/nup.70115

**Published:** 2026-09-08

**Authors:** Tove Stenman

**Affiliations:** ^1^ Department of Health Sciences, Nursing Science Mid Sweden University Östersund Sweden

**Keywords:** capability, confidential conversations, narrative identity, nurse–patient relations, nursing philosophy, palliative care, vulnerability

## Abstract

Confidential conversations constitute an important, still insufficiently explored, aspect of palliative care. While described as valuable in clinical practice, their relational and existential significance remains difficult to conceptualise. This article examines confidential conversations through a philosophical dialogue between empirical insights from specialised palliative care and Paul Ricœur's philosophy of Homo Capax. Ricœur is not applied as a theoretical framework; rather, confidential conversations both illuminate and invite further reflection on his understanding of capability, vulnerability, practical wisdom, solicitude, and narrative identity. The article is based on a philosophical reflection of a synthesis of four qualitative studies conducted in specialised palliative care. It begins from recurring tensions within confidential conversations concerning spontaneity and structure, safety and uncertainty, everyday small talk and existential disclosure, professional responsibility and interpersonal presence. These tensions remain unresolved and are understood as relational conditions through which confidential conversations become possible. Dialogue with Ricœur suggests that confidential conversations can be understood as fragile relational events in which capability and vulnerability are mutually constitutive rather than opposed. This interpretation invites further philosophical reflection on autonomy, asymmetrical vulnerability, and the limits of practical wisdom. To deepen these questions, perspectives from Martin Buber and Hartmut Rosa are brought into dialogue with Ricœur, illuminating how confidential conversations become encounters characterised by responsiveness and resonance while remaining situated within asymmetrical relationships of care. This article suggests that confidential conversations are less understood as communicative interventions or professional techniques than as relational events in which meaning emerges through responsiveness under conditions of suffering, finitude, and institutional constraint. Their significance lies in the possibility that patient and nurse become responsive to one another within a relationship that neither fully controls. The article offers a philosophical interpretation of confidential conversations grounded in empirical experience.

## 1 Introduction

In palliative care, patients live with the awareness that death no longer appears as a distant possibility; it is experienced as an approaching reality (Croker et al. 2022). Living with a life‐limiting illness changes bodily conditions as well as everyday life and relationships (Persson et al. 2020), giving rise to a need to speak about thoughts associated with experiencing time as limited (Yalom 1980). Conversations about death and dying evolve over time as an ongoing, person‐centred process in which end‐of‐life issues are gradually addressed. Within palliative care, conversations acquire particular significance, as what is spoken and what remains unspoken extends beyond the immediate situation towards questions of life and death (Peerboom et al 2023).

Confidential conversations arise within trusting nurse–patient relationships characterised by professional responsibility and openness to experiences that matter deeply to the patient (Davies and Oberle 1990, 1993). Beyond professional obligations of confidentiality, the concept refers to a relational quality through which matters of personal significance become possible to express and receive. Instead of denoting a specific communication technique, confidential conversations describe moments in which patients experience sufficient trust to disclose what matters most to them. Their significance does not reside primarily in what is communicated but in what emerges within the encounter itself. Against this background, confidential conversations appear both important and difficult to conceptualise. Situated within palliative care, whose aim is to relieve suffering and attend to the physical, psychosocial, and existential dimensions of serious illness (World Health Organization 2020), they have long been recognised as central to nursing practice by fostering human connection and emotional support (Davies and Oberle 1990, 1993).

Contemporary healthcare is shaped by biomedical knowledge, institutional routines, and standardised procedures that seek to organise care through predictability and control (Ekman 2022). The conversations patients describe as most meaningful often emerge under conditions that resist prediction and control (Öhlén et al. 2002). These conversations depend upon trust within uncertainty and unfold through forms of responsiveness that cannot be fully prescribed in advance (Höglander et al. 2023).

Trust is central to this process. When patients feel seen and affirmed as persons, they may experience relief from existential suffering (Yalom 1980). Previous research identifies trust as a fundamental condition of caring relationships, enabling presence, authenticity, and engagement between patients and healthcare professionals (Allande‐Cussó et al. 2022; Davies and Oberle 1990, 1993); Höglander et al. 2023). In end‐of‐life care, suffering may undermine confidence in oneself and others, rendering trust simultaneously fragile and necessary (Sacks & Nelson 2007). Rather than eliminating uncertainty, trust enables patients and nurses to remain engaged in conversations whose direction and significance cannot be known in advance. Confidential conversations thus invite a broader question concerning the character of care: how are encounters to be understood when what gives them significance exceeds the logic of intervention?

This article focuses on the moments in which everyday conversation gradually deepens into confidential conversations (Macdonald 2016; Tarbi et al. 2021). Instead of asking how confidential conversations ought to be conducted, the article examines what they disclose about human relating when they occur. It aims to develop a philosophical understanding of confidential conversations as relational encounters by bringing empirical experience into dialogue with Paul Ricœur's philosophy of Ricœur (2011) which understands human beings as simultaneously capable and vulnerable.

## 2 Empirical Point of Departure

This article draws on a synthesis of four situated inquiries conducted within a research programme on confidential conversations in specialised palliative care (Stenman et al. 2023, 2024, 2026a, 2026b). Together the studies illuminate confidential conversations as they are experienced by patients and registered nurses and disclose the relational conditions under which confidential conversations become possible.

The interpreted studies are not presented as new empirical findings. They constitute the experiential basis for a philosophical exploration of confidential conversations. The situations and encounters are approached as expressions of the phenomenon itself, disclosing recurring relational tensions that invite conceptual reflection. These tensions provide the starting point for the dialogue with Ricœur (2011) developed in the following analysis.

## 3 Fragility and Relational Movements in Confidential Conversations

Confidential conversations were experienced as relational processes within the interpersonal encounter, not as communicative events or identifiable interventions. They rarely began as planned conversations about existential concerns. Instead, they developed gradually within ordinary care situations, where everyday small talk shifted into reflections on matters the patient wished or needed to discuss.

Confidential conversations did not follow predictable or uniform patterns. Their emergence depended on patients’ willingness to disclose personal experiences and nurses’ capacity to recognise moments in which disclosure became possible. These encounters unfolded through recurring relational movements, within which confidential conversations gradually took shape. Four recurring movements became visible through which the conversations took shape in specialised palliative care. These were not understood as dichotomies requiring resolution, but as transitions that shaped the conditions under which confidential conversations became possible.

### 3.1 Making Space: Spontaneity and Structure

Confidential conversations rarely arose through scheduled initiatives or predetermined topics. Instead, they could evolve within ordinary nursing care, where routine activities unexpectedly opened into conversations about experiences that mattered deeply to patients. Although these conversations often appeared spontaneous, they were supported by professional efforts to create conditions for their emergence. Nurses described the importance of presence and attentiveness to patients’ verbal and non‐verbal expressions. Structure thus did not produce confidential conversations, but neither was it incidental. It created conditions that supported their development without directing them. These conditions may be understood as necessary but not sufficient for confidential conversations. The nurses created opportunities for conversation to emerge, while whether disclosure occurred depended on the encounter itself, including whether patients judged the moment to be right and the relationship sufficiently safe for personal disclosure.

### 3.2 Entrusting the Encounter: Trust and Uncertainty

A second movement concerned the interplay between trust and uncertainty. Patients described trust as a prerequisite for speaking about deeply personal concerns and emphasised the importance of deciding whether, when, and with whom confidential conversations should take place. For nurses, these conversations were characterised by uncertainty regarding how to respond, what topics might arise, and what emotions might be evoked. Trust did not eliminate uncertainty, nor did uncertainty undermine trust. Trust enabled patients and nurses to remain engaged in conversations whose direction and significance could not be known in advance. Established gradually through continuity, attentiveness, and professional reliability, trust created conditions in which disclosure could become possible without guaranteeing either disclosure itself or mutual understanding.

### 3.3 Everyday Small Talk and Existential Concerns

A third movement concerned the relationship between everyday small talk and existential concerns. Confidential conversations rarely began with explicit reflections on death, meaning, or suffering. Ordinary conversation gradually acquired a different significance, as comments about everyday life opened into reflections on loss, fear, or approaching death without any clear transition between them. Everyday small talk and existential concerns were not separate forms of communication, but intertwined dimensions of the same conversational process. What initially appeared ordinary could gradually take on existential significance within the conversation, creating possibilities for experiences to be expressed that might otherwise have remained unspoken.

### 3.4 Being Present: Professional Responsibility and Interpersonal Presence

A fourth movement concerned the relationship between professional responsibility and interpersonal presence. Confidential conversations took place within established nurse–patient relationships and were inseparable from the responsibilities inherent in professional care. At the same time, patients valued nurses who were personally present while remaining professionally grounded. Nurses described the challenge of responding as fellow human beings without losing sight of their professional responsibilities. Remaining present in situations marked by suffering did not necessarily involve providing answers or solutions, but staying with the patient within uncertainty. Professional responsibility and interpersonal presence were therefore not experienced as competing demands. Confidential conversations took shape through their interplay, where professional responsibility was realised through the quality of the relationship and not through communicative techniques alone.

Taken together, the four movements suggest that confidential conversations cannot readily be understood through familiar distinctions such as planned or spontaneous, professional or personal, and everyday or existential. These dimensions remained intertwined within the encounter.

## 4 Conceptual Framing: Ricœur as Interpretive Dialogue

Confidential conversations in palliative care resist straightforward conceptualisation. Instead of presenting predefined topic of communication, they unfold through shifting configurations of speech, silence, timing, and affect. Meaning is not simply transmitted but develops within the encounter itself. Confidential conversations resist reduction to either technique or structure. They are neither fully intentional nor fully contingent. What appears is an interpersonal encounter in which what can be said is continuously shaped by the fragile openness of the situation.

Ricœur (2011) is approached as a philosophical dialogue partner whose concepts provide a language for deepening understanding of the phenomenon. Through concepts such as capability, vulnerability, practical wisdom, solicitude, and narrative identity, the dialogue seeks to illuminate dimensions of confidential conversations while remaining attentive to the questions that arise when these concepts are brought into dialogue with the realities of palliative care.

### 4.1 Homo Capax: Capability and Vulnerability

Homo Capax (Ricœur 2011) illuminates the intertwining of capability and vulnerability that characterises confidential conversations. A person is understood as both capable and vulnerable; capability does not exist despite vulnerability but through it. The capacity to speak and respond presupposes openness to being affected by others. Capability and vulnerability therefore remain inseparable and mutually constitutive dimensions of personhood (Ricœur 2011).

In confidential conversations, this understanding becomes visible. Patients and nurses described conversations in which experiences could be shared within relationships shaped by trust and responsiveness. What became possible in these conversations depended not only on the capacity to speak and listen, but also on the openness to share and be affected by what was shared. From this perspective, confidential conversations can be understood as interpersonal encounters between capable and vulnerable persons. The capacities to speak, listen, and respond emerge through the relationship itself rather than independently of it.

Homo capax (Ricœur 2011) draws attention to how capability and vulnerability remain intertwined within the conversation. Confidential conversations thus invite further reflection on how this intertwining is lived and expressed under conditions shaped by temporality, institutional organisation, and asymmetrical relationships.

Trust within confidential conversations is not always directed solely towards a particular individual. It may also be grounded in the professional relationship the nurse embodies. Confidence in the professional responsibilities and ethical obligations of the nursing role can create conditions in which experiences of vulnerability may be expressed and shared. In Ricœur's terms, trust can be understood as grounded in the tension between human capability and vulnerability, allowing engagement with others despite uncertainty and the limits of one's control over outcomes.

### 4.2 Practical Wisdom and Solicitude

Ricœur (2011) reinterprets the concept of phronesis (practical wisdom) that becomes relevant in situations where action cannot be derived from rules, and where ethical judgement must be enacted under conditions of uncertainty. Confidential conversations illuminate these situations: nurses orienting themselves within encounters that are not pre‐structured, and where the meaning of silence, disclosure, or withdrawal is not given in advance.

These encounters indicate that practical wisdom is not simply a matter of individual judgement. It is exercised within relational and institutional conditions that shape what counts as an appropriate response. Timing, continuity, workload, and organisational logics are not external to judgement; they are part of its conditions of possibility.

Ricœur's (2011) description of solicitude clarifies the relational orientation of these encounters, particularly the recognition of the other as both vulnerable and capable. Within the confidential conversations, this recognition is not a stable stance but an ongoing negotiation. Nurses do not occupy a position outside the relation from which they apply responsiveness; they are already inside it, affected by it, and partially constituted through it. This complicates any clear separation between ethical competence and relational exposure.

### 4.3 Narrative Identity

Narrative identity describes identity as configured through temporal interpretation, not as a fixed substance (Ricœur 2011). Confidential conversations both resonate with and destabilise this claim; they rarely take the form of coherent narrative constructions. Instead, they appear as fragments: interrupted statements, partial articulations, shifts between registers of meaning, and silences that resist integration. These fragments are not deficiencies of narrative; they indicate that narrative configuration may remain incomplete without thereby losing significance.

Meaning emerges through the relational reception of fragments that need not form a coherent life story. Narrative identity is therefore co‐constituted in encounters that are temporally and institutionally constrained (Kristensson Uggla 2022). Confidential conversations illuminate a relational dimension of narrative identity in which meaning may remain suspended across encounters, carried by fragments that render aspects of lived experience temporarily shareable. The professional character of the relationship may also contribute to this openness. Within confidential conversations, patients are not necessarily required to sustain familiar roles or coherent self‐understandings. The encounter can therefore create conditions in which uncertainty and fragmentation are expressed without immediate pressure towards narrative integration. Meaning may emerge through what remains unfinished as much as through what becomes coherent.

## 5 Dissonance: Continuing the Dialogue

The preceding discussion has shown how Ricœur's concepts illuminate features of confidential conversations in palliative care. The conversations also bring into view aspects of these encounters that are not fully captured by Ricœur's conceptual vocabulary. These may be understood as productive tensions emerging within the dialogue between philosophical concepts and lived experience.

### 5.1 Autonomy Reconsidered

Patients described the importance of deciding if, when, how, and with whom they wished to engage in confidential conversations. At first sight, this may appear to affirm autonomy, yet autonomy alone does not capture what was at stake. What proved decisive was not merely the capacity to choose, but whether conversation became possible within relationships characterised by trust and recognition. These were not external conditions for an already autonomous act; they were conditions through which speech could take form. Patients’ descriptions of confidential conversations suggest that the capacity to speak and disclose is exercised within a relational context that neither participant fully controls.

When vulnerability is conceived primarily as a threat to autonomy, the relation between them remains external (Hettema 2014). Capability is understood through vulnerability rather than in opposition to it. The capacities to speak and respond are situated within relations that both enable and expose the self. Confidential conversations make this interdependence visible. This relational understanding is relevant in palliative care, where profound suffering is a feature of patients’ experiences (Rattner 2020). For Ricœur (1992), suffering involves a diminution of the capacity to act and a disruption in the relation between self and other. It therefore challenges an understanding of the person as an autonomous and self‐sufficient subject and draws attention to the sustaining significance of relations with others. Under these circumstances, the significance of confidential conversations extends beyond autonomous decision‐making. They provide a space in which vulnerability can be acknowledged and endured.

Understanding vulnerability as a condition of capability allows autonomy to be understood differently. While nursing ethics has emphasised respect for patient autonomy partly in response to paternalistic traditions (Woodward 1998), care ethics has questioned whether an excessive emphasis on autonomy risks obscuring forms of dependency that are constitutive of human existence (Verkerk 2001). Ricœur offers a way of understanding autonomy that resonates with concerns from both traditions. Autonomy remains ethically significant, though not as independence from vulnerability. Instead, it can be understood as a capability exercised through relations of care.

Confidential conversations highlight that capability depends on relations that expose individuals to conditions beyond their control. Autonomy is not understood as sovereign self‐determination. Hettema (2014) similarly argues that Ricœur does not reject autonomy, as he reconfigures it as intrinsically vulnerable rather than independent. From this perspective, confidential conversations are less practices aimed at strengthening autonomy than encounters through which vulnerability becomes speakable. Responsiveness to others is not a limit to action but one of the conditions through which human capability is exercised.

### 5.2 Asymmetrical Vulnerability and Mutual Affectivity

Confidential conversations reveal vulnerability across three interconnected levels: as an existential condition, a relational experience, and a structural asymmetry. These distinctions are important for understanding how mutual affectivity can arise within the conversation that remain fundamentally unequal.

From an existential perspective, vulnerability is a shared feature of human existence. Within Ricœur's (2011) philosophy, vulnerability is inseparable from capability and relational existence. Both patients and nurses are capable and vulnerable persons whose lives may be affected by what takes place in the encounter. At this level, vulnerability does not belong exclusively to either party but characterises the human condition itself.

In clinical practice, however, this shared vulnerability is lived under asymmetrical conditions. The patient is situated in a condition marked by illness and dependence. While vulnerability characterises human existence, dependency refers to situations in which vulnerability requires the care of specific others (Dodds 2014). Dependency does not replace capability but reveals its relational and fragile character (Ricœur 2011). The patient's opportunities to participate in and influence care are therefore shaped by relational and organisational conditions, reflecting what Ekman (2022) describe as a threefold subordination comprising existential, epistemic, and institutional dimensions.

The nurse's vulnerability is configured differently. It is shaped by exposure to suffering and by the responsibility to remain professionally available within institutional constraints. As Yalom (1980) notes, encounters with dying persons involve a confrontation with one's own mortality, making the encounters existential as well as professional. Nevertheless, the nurse does not occupy the same position as the patient. The vulnerabilities of patient and nurse are therefore not equivalent, even though both may be affected by what occurs in the encounter.

The relational level helps explain how mutual affectivity remains possible despite this asymmetry. Both nurses and patients described confidential conversations as opportunities to disclose concerns that were not readily voiced elsewhere. The possibility of speaking depended upon trust and responsiveness. What mattered was the presence of someone willing and able to listen. Vulnerability was experienced not only as exposure but also as a need for recognition and response. This finding points to an important distinction between asymmetry and mutuality. As Ekman (2022) argue, caring relationships are inherently asymmetrical, shaped by differences in dependency, knowledge, and responsibility. The ethical challenge is therefore not to eliminate asymmetry but to understand how reciprocity may arise within it. Confidential conversations suggest that although patients remain dependent and nurses professionally responsible, these conditions do not fully determine the encounter. Moments of disclosure may open possibilities for recognising one another as persons rather than solely as patient and healthcare professional.

Care ethics has similarly shown that institutional arrangements may both constrain and enable relational engagement (Verkerk 2001), while empirical studies demonstrate how organisational conditions may undermine or support person‐centred encounters (Phelan et al. 2021). Confidential conversations appear significant because they create opportunities for recognition within institutional settings. The analytical question, then, is not whether vulnerability is shared in an identical sense, but how persons affect one another under unequal conditions. Vulnerability is neither uniform nor symmetrically distributed. If reduced to a general condition of shared vulnerability, the specificity of dependency and responsibility is obscured (Dodds 2014). If understood solely through asymmetry, the reciprocity that remains possible within caring relationships risks being overlooked. The concept of solicitude (Ricœur 2011) points towards a form of mutuality grounded in responsiveness to the other. Mutual affectivity therefore does not require symmetry. What is shared is not vulnerability in an identical sense, but the human capacity to be affected by another's vulnerability and to respond within relationships that remain structurally unequal.

### 5.3 The Encounter and Resonance

Confidential conversations unfold in ways that cannot be fully anticipated or controlled. Silence acquires significance, familiar patterns of interaction may shift, and the character of the relationship itself can change. What is at stake is not a failure of communication but a transformation in how participants encounter one another.

Practical wisdom provides a resource for understanding ethical responsiveness under conditions of uncertainty (Ricœur and Backelin 2011). Confidential conversations nevertheless reveal a tension between role and relationship that points towards dimensions of the encounter exceeding deliberation and professional competence alone. Patients seek not only professional competence but also human presence, while nurses describe the challenge of remaining responsive to another person's suffering without relinquishing professional responsibility. The professional structure of care remains intact, yet the encounter may acquire a different character. In those moments, the other appears simultaneously as patient and as fellow human being.

This recognition does not dissolve the professional relationship. On the contrary, the possibility of confidential conversations may depend upon it. Patients may seek conversations because nurses occupy a professional role characterised by responsibility and confidentiality. Within the relationship, patients need not carry the same responsibility for the emotional consequences of disclosure as they might in other relationships. Professionalism need not constrain confidential conversations; it can provide conditions that make them possible. Professional restraint may itself constitute a form of responsiveness, preserving openness to the other's experience while allowing the patient's concerns to remain at the centre of the encounter.

Buber (1994) distinction between *I–It* and *I–Thou* relations offers one way of articulating this shift. The distinction concerns not the formal structure of the relationship but the mode in which the other is encountered. Within an I–It relation, the other is approached primarily through the purposes that organise the encounter, whether this concern care, communication, or professional responsibility. An I–Thou relation becomes possible when the other is encountered as a person whose significance exceeds determinations. What changes is therefore not the disappearance of professional structure, but the possibility of encountering the other in ways that are not fully reducible to it.

Rosa's concept of resonance illuminates the experiential quality of these encounters (Rosa 2019). Resonance refers to a responsive relation in which persons are affected by what takes place between them and respond in ways that cannot be fully anticipated or controlled. Confidential conversations may become moments in which patient and nurse are drawn into a shared responsiveness to what unfolds between them. The significance of the encounter derives less from what is accomplished than from the responsive connection that develops between participants. Resonance therefore helps explain how a professional encounter can become deeply human without ceasing to be professional.

What is disclosed is not friendship, but a form of human connection in which professional responsibility and interpersonal responsiveness become inseparable (Antonytheva et al. (2021). The patient remains a patient, and the nurse remains a nurse, yet the significance of each exceeds institutional role descriptions (Cribb et al. 2021). Resonance thus neither dissolves nor simply reproduces professional roles. It illuminates how meaningful encounters may arise within them (Rosa 2019).

This also elucidates the distinction between authentic responsiveness and procedural enactment (Ekman 2022). Professional knowledge can guide decisions concerning presence and silence, and communicative competence can support meaningful interaction (Öhlén & Friberg 2023). What neither can guarantee, however, is resonance. A response organised primarily through communicative strategy may reproduce the outward appearance of attentiveness while lacking the openness through which resonance becomes possible. The distinction lies less in what is done than in the quality of the relationship through which it is done (Rosa 2019).

The fragility of these encounters should not be overlooked. Nurses can create conditions favourable to dialogue, but neither disclosure nor resonance can be produced. Meaningful encounters may occur or fail to occur without being reducible to individual success or failure (Öhlén & Friberg 2023). Confidential conversations remain situated within a field of realised and unrealised possibilities, where absence may be as analytically significant as presence. Resonance thus designates a contingent form of relational vitality. It cannot be secured through technique or ethical design. Its significance lies in its resistance to control. In this sense, responsiveness may also take the form of non‐intervention, insofar as it sustains openness to the other without reducing the encounter to action.

## 6 Institutional Implications: Conditions of Relational Palliative Care

An institutional dimension must be considered that cannot be reduced to individual encounters. Healthcare organisations are structured by demands for documentation, measurability, and efficiency. These frameworks are necessary for the functioning of care, yet they also shape the conditions under which relational encounters become possible. In palliative care this raises a particular challenge, since significant dimensions of suffering do not present themselves as problems that can be solved (Rattner & Berzoff, 2016; Rattner 2020). Experiences of loss and finitude exceed the logic of intervention that structures much of contemporary healthcare. This does not indicate a failure of care but points to limits inherent in understandings of care primarily oriented towards intervention (Rattner 2019; Rattner and Cait 2024).

Healthcare systems derive legitimacy from their capacity to intervene and evaluate (Ekman 2022). Palliative care, however, repeatedly confronts situations in which the central question is no longer what can be done, but how one remains present when little can be changed (Croker et al. 2022). Documentation and evaluation remain necessary because they support accountability and coordination. At the same time, they privilege forms of knowledge centred on intervention and outcome, while dimensions of care grounded in presence and attentiveness become more difficult to render visible (Phelan et al. 2021).

Confidential conversations exist within a field of realised and unrealised possibilities. Encounters are interrupted, postponed, or never initiated and absences cannot be understood solely as individual shortcomings. They reflect how care is organised and what forms of practice become possible within that organisation (Ekman 2022). Confidential conversations therefore raise broader questions about how organisational conditions shape what becomes visible, meaningful, and worthy of attention. When focus is directed primarily towards interventions and measurable outcomes, forms of care that do not produce observable change risk becoming less recognisable within practice.

For the dying person, suffering could confront realities that cannot be resolved (Yalom 1980). Under these circumstances, the impulse to intervene may itself become a way of distancing oneself from vulnerability. Learning to remain present in silence therefore represents more than a communicative skill (Lagerin et al. 2025). It expresses a willingness to encounter dimensions of suffering that resist intervention. What is at stake is not inactivity, but a mode of professional presence that does not equate care with action (Rattner and Berzoff 2016; Rattner 2020).

Two understandings of care come into view. One is oriented towards intervention, evaluation, and measurable outcomes. The other begins from the recognition that some dimensions of human suffering cannot be repaired and that care may consist in remaining present to what cannot be changed. Confidential conversations illuminate the tension between these understandings without resolving it.

## 7 Conclusion

This article has examined confidential conversations in palliative care not as communicative techniques or professional interventions, but as relational events. Bringing empirical accounts into dialogue with Ricœur's philosophy has shown how concepts such as capability, vulnerability, practical wisdom, solicitude, and narrative identity illuminate central dimensions of these encounters. The conversations also point to dimensions of the encounter that are not fully captured by Ricœur's conceptual vocabulary alone.

Confidential conversations do not merely represent the application of ethical competence; they disclose forms of relating that cannot be fully anticipated or governed. Buber's account of the I–Thou relation clarifies the character of these encounters, while Rosa's concept of resonance captures their contingent and responsive quality. Resonance is not positioned as an alternative to Ricœur's ethics; it extends the analysis by illuminating relational qualities that exceed deliberative judgement.

Confidential conversations may be understood as fragile relational events. Their significance lies less in information exchange, autonomous decision‐making, or professional competence than in the possibility that patient and nurse become responsive to one another within an irreducibly asymmetrical relationship shaped by finitude. This perspective shifts attention from care as the implementation of interventions towards care as a relational practice in which ethical judgement, vulnerability, and responsiveness remain inseparable from the conditions that make encounter possible.

## Author Contributions

**Tove Stenman:** conceptualization, data curation, formal analysis, investigation, methodology, project administration, validation, visualization, writing – original draft, writing – review and editing.

## Funding

The author has nothing to report.

## Ethics Statement

All four qualitative sub‐studies on which this synthesis is based were conducted in accordance with ethical guidelines for research involving humans (Swedish Research Council, 2017), the Declaration of Helsinki (WMA, 2024), and the *ICN Code of Ethics for Nurses* (ICN, 2021). Each study received ethical approval from the Swedish Ethical Review Authority (Reference numbers: 2021‐04066; 2022‐03769‐02; 2024‐00341‐02). Detailed ethical procedures are reported in the original publications. The present manuscript involves a secondary, integrative synthesis of previously collected and ethically approved qualitative material. No new data were generated, and no identifiable information was accessed beyond what had already been de‐identified in the original studies. Data handling complied with the General Data Protection Regulation (GDPR; SFS 2018:218), and all material used for the synthesis was stored and processed in coded and anonymised form.

## Conflicts of Interest

The author declares no conflict of interest.

## Data Availability

The datasets used and/or analysed during the current study are available from the corresponding author upon reasonable request, provided that no sensitive, personal, or confidential data are disclosed. The data are stored on a password‐protected server at Mid Sweden University.
